# Factors Influencing Employees’ Subjective Wellbeing and Job Performance During the COVID-19 Global Pandemic: The Perspective of Social Cognitive Career Theory

**DOI:** 10.3389/fpsyg.2021.577028

**Published:** 2021-03-03

**Authors:** Tzai-Chiao Lee, Michael Yao-Ping Peng, Lin Wang, Hao-Kai Hung

**Affiliations:** ^1^Department of Accounting and Auditing, Guangxi University of Finance and Economics, Nanning, China; ^2^School of Economics & Management, Foshan University, Foshan, China; ^3^Department of Business Administration, Yango University, Fuzhou, China

**Keywords:** prior knowledge, perceived organizational support, self-efficacy, employee employability, subjective well-being, job performance

## Abstract

The novel coronavirus disease that emerged at the end of 2019 began threatening the health and lives of millions of people after a few weeks. However, social and economic problems derived from COVID-19 have changed the development of individuals and the whole country. This study examines the work conditions of Taiwanese versus mainland China employees, and evaluates the relationship between support mechanisms and subjective wellbeing from a social cognitive career theory perspective. In this study, a total of 623 Taiwanese questionnaires and 513 mainland China questionnaires were collected to compare the two sample groups in terms of the development of employees’ subjective wellbeing. The results show that the Taiwanese sample had more significant development paths compared to mainland China employees in terms of prior knowledge, perceived organizational support, self-efficacy, employee employability, subjective wellbeing, and job performance. Finally, based on the conclusions, this study proposes some specific suggestions on theoretical mode for future studies.

## Introduction

The influence of the work environment and employability of employees on job satisfaction and job performance (JB) has always been a key focus in the field of human resource management (Brown et al., 2011; Chang and Edwards, 2015; Kurtessis et al., 2017; Akgunduz et al., 2018). Many studies have found that a good work environment will help employees strengthen their work efforts and acquire knowledge and the skills they need (Lent et al., 2011; Lamm et al., 2015; Liguori et al., 2019), thus improving the psychological state of achieving the goals they set (Chin and Rasdi, 2014; Duffy et al., 2014; Hajizadeh and Zali, 2016). Most of these studies were conducted in a complete work environment (Lent et al., 2011; Ahmed and Nawaz, 2015; Lamm et al., 2015; Akgunduz et al., 2018; Liguori et al., 2019). In particular, a majority of these studies have verified the importance of online meetings or SNS advisors. However, since the global pandemic of COVID-19 started in January 2020, countries all over the world have begun to stop exchanges, such as economics, tourism, and production, especially work activities. In order to contain the spread of COVID-19, countries have had to cease many economic activities, and extend the halted production restart date. In order to enable employees to continue working in the process of combating the epidemic, employees have started to work online at home (Vaziri et al., 2020), which allows employees to gain salary with the help of technological carriers. Such sudden changes in job category (Kramer and Kramer, 2020) bring job insecurity to employees (Blustein et al., 2020). For crisis management in response to the pandemic (Eby et al., 2016), there will be different understandings and reactions based on employees’ cognitive differences in factors such as organizational and external environment (Morgeson et al., 2015). Nevertheless, the impact of employees’ acceptance of online working within an inadaptable work environment on JB remains to be observed (Lamm et al., 2015; Thompson et al., 2017; Akgunduz et al., 2018), especially because everyone in such an uncertain context feels anxious and stressed (Chang and Edwards, 2015; Schultz et al., 2015; Jemini-Gashi et al., 2019). Therefore, this study intends to explore the current development status of employee working activities in the context of the COVID-19 global pandemic.

A majority of studies on organizational behavior have discussed factors influencing the working outcomes of employees (Lent et al., 2011; Ahmed and Nawaz, 2015; Chang and Edwards, 2015; Liguori et al., 2019), or the application effect of working factors (Caesens and Stinglhamber, 2014; Akgunduz et al., 2018). Some studies in recent years began to discuss the shape of employee subjective wellbeing (SWB) from the view of organizational psychology (Lent et al., 2011; Duffy et al., 2014; Chang and Edwards, 2015; Thompson et al., 2016; Kurtessis et al., 2017). The emergence of positive psychology leads psychology into a new direction (Lent et al., 2011; Liguori et al., 2019). Under the influence of positive psychology, counseling and psychotherapy began to turn their attention to positive affect subjects (Ahmed and Nawaz, 2015; Akgunduz et al., 2018). Many scholars advocate for the emphasis of wellbeing in adolescents (Thompson et al., 2016), and believe that SWB is the core of mentally healthy development in adolescents (Lent et al., 2011; Schultz et al., 2015; Kurtessis et al., 2017). This study emphasizes employee SWB as the core view: (1) SWB, as the major concern of employee personality and social psychology, is used to examine the social change and improvement of organizational policies and solve employees’ working problems (Thompson et al., 2016; Kurtessis et al., 2017; Meyers et al., 2019); (2) The discussion of employee SWB will put emphasis on finding symptoms such as possible depression, anxiety, and psychological disorders (Schultz et al., 2015); the positive and negative psychology lies between two extremes of continuous psychological states (Kurtessis et al., 2017), and better SWB of employees will help employees face challenges with a positive psychological state, and increase the value of JB (Gillet et al., 2012; Caesens and Stinglhamber, 2014; Thompson et al., 2016). Considering the above reasons, this study aims to further understand and discuss the development course of employee SWB through enhancing EE in the psychological enhancement process (Kurtessis et al., 2017). (3) From the angle of cross-culture, it can be seen that there are similar measurements for JB across different cultures, but in terms of SWB, Western culture upholds individual feeling and independence (Rehg et al., 2012; Meyers et al., 2019), while oriental culture puts emphasis on social norms and the value of sharing and co-fusion (Schultz et al., 2015). Western and oriental cultures also have varied ways of understanding, experiencing, and pursuing wellbeing. Based on the above reasons, this study aims to explore the development of employees’ SWB in changing working activities.

The social cognitive career theory (SCCT) contributes to building an appropriate research framework to discuss the relevance between working activities, environmental influencing factors, and psychological needs (Brown et al., 2011; Chin and Rasdi, 2014; Duffy et al., 2014; Chang and Edwards, 2015; Jemini-Gashi et al., 2019). According to the SCCT, Lent et al. (2002) hold that personal attribution, environmental influencing factors, and intentional behaviors form a triangular relationship of interaction (Lent et al., 2011; Caesens and Stinglhamber, 2014; Lamm et al., 2015; Jemini-Gashi et al., 2019; Liguori et al., 2019; Meyers et al., 2019). In other words, individual behaviors are formed by the interaction of individual’s inner thoughts, emotions, and environment (Brown et al., 2011; Chin and Rasdi, 2014; Duffy et al., 2014; Chang and Edwards, 2015). It is found from the SCCT architectural pattern that there is an indirect effect of personal cognitive factors between environmental factors and behavioral factors (Lent et al., 2011; Duffy et al., 2014; Thompson et al., 2016; Jemini-Gashi et al., 2019; Liguori et al., 2019). In other words, when personal cognitive factors are expected to directly affect employees’ SWB (Ahmed and Nawaz, 2015; Hajizadeh and Zali, 2016; Kurtessis et al., 2017), the effect of external environmental factors on employees’ SWB becomes negligible (Schultz et al., 2015; Thompson et al., 2016; Liguori et al., 2019; Meyers et al., 2019). Self-efficacy is not only the belief of employees in their own successful performance and specific behaviors and abilities related to organization (Brown et al., 2011; Chang and Edwards, 2015), but also an important factor inspiring spontaneous work involvement and engagement (Caesens and Stinglhamber, 2014), as well as the core of SCCT (Lent et al., 2011; Thompson et al., 2016; Sheu and Bordon, 2017; Jemini-Gashi et al., 2019; Liguori et al., 2019). Thus, this study proposes that the combination of cognitive factors and the SCCT between self-efficacy and employees’ SWB will enrich the existing literature.

Moreover, in the aspect of individual cognitive factors, when employees perceive the expectation and affirmation of people important to them, they will perform better (Lent et al., 2011; Duffy et al., 2014; Hajizadeh and Zali, 2016; Liguori et al., 2019). Scholars have found that the interaction of employees with people they find important such as supervisors and peers will have an effect on their career interests and JB (Brown et al., 2011; Duffy et al., 2014; Ahmed and Nawaz, 2015; Chang and Edwards, 2015; Lamm et al., 2015; Akgunduz et al., 2018). Because of the profound implications form both individual and organizational factors (Cordova et al., 2014; Chang and Edwards, 2015), this study proposes that prior knowledge (PK) (Ineson et al., 2013; Williams and Lombrozo, 2013; Li et al., 2015; Hajizadeh and Zali, 2016) and perceived organizational support (Caesens and Stinglhamber, 2014; Ahmed and Nawaz, 2015; Kurtessis et al., 2017; Akgunduz et al., 2018; Meyers et al., 2019) (POS) are important individual and organizational cognitive factors in the enhancement process of employees’ skill, and employability is the enhancement output (Chin and Rasdi, 2014; Cordova et al., 2014; Chang and Edwards, 2015; Akgunduz et al., 2018; Jemini-Gashi et al., 2019; Liguori et al., 2019). This includes the development of employees for employment, the enhancement of their employability, and so forth (Ineson et al., 2013; Akgunduz et al., 2018). Regarding the psychological and sociological characteristics, this study is based on employees’ PK and POS (Ineson et al., 2013; Williams and Lombrozo, 2013; Cordova et al., 2014; Ahmed and Nawaz, 2015; Chang and Edwards, 2015; Li et al., 2015; Jemini-Gashi et al., 2019). The employees’ PK and POS influence employees’ employability (EE), so that both factors are the most important resources for employees to gain more self-efficacy and enhance their EE.

According to the report of Bloomberg in March 2020, only employees in Taiwan and Sweden attend to work as usual due to the global pandemic of COVID-19. Isolation policies of different levels have been implemented in each country in response to the severity of the pandemic, thus leading to significant differences in the economic operation of different regions. Even in the same country, there may be different policies of isolation, making people’s life, work, and interaction different. For instance, there are differences in policies of isolation for people entering and leaving in Hubei Province and Hainan Province in mainland China. In order to explore the differences of regions in working activities caused by environmental threat factors and the changes of employee SWB (Schultz et al., 2015) in mainland China and Taiwan, research samples were taken of interregional comparison in order to learn about the relevance of the research variables (Hansen et al., 2012; Rehg et al., 2012; Meyers et al., 2019). Therefore, this study focuses on determining employees’ perceptions of the individual and organizational drivers of EE, self-efficacy, SWB, and JB in an organization, as well as the relationships among them (Ahmed and Nawaz, 2015; Akgunduz et al., 2018). The following questions are investigated:

(1) Are there significant associations among employees’ perceptions of PK, POS, self-efficacy, EE, SWB, and JB?
(2) Do EE and self-efficacy play mediating roles in the relationship between the antecedents (individual and organizational drivers) and consequences of SWB?
(3) Due to the global pandemic of COVID-19, do various working activities influence the effect of employees’ working antecedents on self-reported gains in SWB?

## Literature Review and Hypotheses Development

### Theoretical Background of Social Cognitive Career Theory

Social Cognitive Career Theory (SCCT) is used as an initial foundation in this study for effective POS towards sustainable employees’ competence enhancement and wellbeing (Lent et al., 2011; Chang and Edwards, 2015; Jemini-Gashi et al., 2019; Liguori et al., 2019). SCCT is an empirically validated model that has been widely accepted (Brown et al., 2011; Chin and Rasdi, 2014; Duffy et al., 2014). It is a method for understanding and predicting changes in human behaviors and cognitive behaviors. According to this theory, human meta-development occurs through continuous interaction with the external environment, and the environment must go through a cognitive process before affecting human behaviors (Lent et al., 2011; Duffy et al., 2014; Chang and Edwards, 2015; Thompson et al., 2016; Liguori et al., 2019). The theory proposes that there is a ternary interactive and causal relationship between cognitive factors, environmental factors, and human behaviors (Brown et al., 2011; Chang and Edwards, 2015; Hajizadeh and Zali, 2016; Akgunduz et al., 2018). Behavior is influenced by both cognitive and environmental factors. Specifically, cognitive factors refer to individual’s cognition, emotion, and actual events, and environmental factors refer to the social and physical environments that can affect human behaviors (Brown et al., 2011; Lent et al., 2011; Chin and Rasdi, 2014; Chang and Edwards, 2015).

According to Lent et al. (1994), self-efficacy is the key structure of SCCT and is believed to have a direct impact on behavior (Brown et al., 2011; Duffy et al., 2014; Chang and Edwards, 2015; Liguori et al., 2019). The outcome expectation is the second structure of SCCT, representing a person’s judgment on the consequences resulting from the execution or non-execution of a specific behavior (Brown et al., 2011; Caesens and Stinglhamber, 2014; Chin and Rasdi, 2014; Duffy et al., 2014). The pattern of manifestation of outcome expectation can be embodied as self-perception such as SWB (Thompson et al., 2016; Kurtessis et al., 2017). The goal is the third core structure of SCCT, and can have a direct impact on behavior and regulate other structures in the model. Achievement of goals requires specific self-regulation skills, such as gaining EE and completing specific goals (Brown et al., 2011; Lent et al., 2011; Caesens and Stinglhamber, 2014; Duffy et al., 2014).

Although Lent et al. (1994) clearly described a social cognitive career structural network, self-efficacy in the past studies has received more attention than other model groups or only one or two other variables are used to examine self-efficacy (Brown et al., 2011; Duffy et al., 2014; Chang and Edwards, 2015). This study believes that self-efficacy cannot be studied in isolation (Lent et al., 2011; Caesens and Stinglhamber, 2014; Jemini-Gashi et al., 2019; Liguori et al., 2019). We will use the SCCT framework to further understand the impact of changes in the working environment of employees in mainland China and Taiwan during the global epidemic of COVID-19 on SWB (Hansen et al., 2012; Chang and Edwards, 2015; Thompson et al., 2016; Kurtessis et al., 2017). More specifically, the purpose of this study is to examine the impact of PK and POS on self-efficacy and EE, analyze the relationship with employees’ SWB, and determine whether the effect arising from such a relationship varies within regions (Sheu and Bordon, 2017).

### Subjective Wellbeing (SWB)

People will eventually begin to reflect on the self-seeking mode of material satisfaction, further seek psychological satisfaction, and begin to emphasize the importance of quality of life (Kurtessis et al., 2017); thus the importance of the proposal of the concept of SWB (Hanson et al., 2016; Denovan and Macaskill, 2017; Stallman et al., 2018). SWB is a result of satisfaction of life coupled with perceived positive and negative emotional intensity (Schultz et al., 2015; Evans et al., 2017). Keyes and Waterman (2003), Keyes (2005) expanded the definition to incorporate the concept of “social wellbeing” by merging the two (psychological wellbeing and emotional wellbeing) to delineate SWB as a sum of three aspects: in the sense of psychological wellbeing (Kurtessis et al., 2017; Meyers et al., 2019), it serves to explore self-psychological adjustment and the macro-consciousness of the individual’s inner self; a sense of evaluating the function of the self in life through public and social norms; and lastly, emotional wellbeing as the individual’s awareness and assessment of the emotional state of self-life (Gillet et al., 2012; Evans et al., 2017). Under the great social pressure brought on by COVID-19, enterprises need to pay more attention to employees’ SWB to enhance employees’ tenacity, which is the spiritual guarantee for the organization to resume operation and promote the sustainable development of enterprises (Carnevale and Hatak, 2020).

For a long time, employees in the working environment have faced many psychological and physical pressures that cause employees to fail when handling learning challenges with a positive attitude (Ahmed and Nawaz, 2015). Bewick et al. (2010) point out, in a study taking British employees as the research object, that employees often have considerable pressures of loans, life, and performance compared with their peers and emphasize that scholars should shift their focus from working performance to the discussion of the psychological problems of employees (Kurtessis et al., 2017; Meyers et al., 2019). Although scholars have discussed employee SWB from different levels, there are still some research gaps that are worth discussing and exploring, such as how SWB develops (Gillet et al., 2012), and internal and external factors that affect employees’ mental health and SWB (Ahmed and Nawaz, 2015). In addition, Folkman and Moskowitz (2000) point out in their research that future research should focus on the discussion of positive emotions and SWB (Kurtessis et al., 2017), because it is impossible to find relevant factors that can effectively reduce mental health problems derived from stress if it is not discussed from the perspective of positive outcomes (Thompson et al., 2016). Therefore, based on the SCCT, this study uses SWB as the outcome variable to explore the influence of relevant factors on it. This means that in the context of the COVID-19 pandemic, the positive psychology of SWB gives employees a sense of security, makes them settle down in the job, and improves JB. Thus, this study proposes H1 as follows:

*H1: SWB has a positive and significant impact on employees’ JB.*

### Employee Employability (EE)

In recent years, scholars have put more effort into employability-related research (Ineson et al., 2013; Thompson et al., 2016). The substantial technological, social, and economic changes that have occurred in recent decades have modified the concepts and operations of industrial organizations across the world (Abbas et al., 2015; Akgunduz et al., 2018; Abbas and Sağsan, 2019). Hence, dynamic organizations ensure the highest standards of human capital development, so that they can contribute to economic growth (Ahmed et al., 2015; Baek and Cho, 2018). Through research situations and the design of methods, and the integration of theoretical and practical analysis (Ineson et al., 2013), scholars have studied the meaning of EE and the causality between EE and other factors (Hennemann and Liefner, 2010; Thompson et al., 2016; Baek and Cho, 2018). Heijde and Van Der Heijden (2006) have argued that EE is the individual’s appropriate application of competence (Pan and Lee, 2011; Blázquez et al., 2018), continuous acquisition and creation of essential work skills in order to accomplish all the tasks, and adaptation to internal and external labor market changes (Chang and Edwards, 2015; Akgunduz et al., 2018). Hence, the need for critical and reflective thinking, problem-solving abilities, self-management, learning, and related competencies is continually increasing across all disciplines (Ineson et al., 2013; Thompson et al., 2016; Makkonen and Olkkonen, 2017). Several prior studies have indicated that in addition to the influence of basic education on EE, factors like personal conditions, interpersonal relationships, and external factors that cannot be acquired in human resources should also be considered. Pan and Lee (2011) surveyed the samples in Taiwan, adopting the employability scale developed by Andrews and Higson (2008). They suggested that employability should cover the general and professional ability required at work, work attitude, career planning ability, and confidence. This study adopts the employability classification of Pan and Lee (2011) as the measure of EE.

De Cuyper et al. (2008) considers EE as having its importance in post-industrial knowledgeable society by continuously updating knowledge to maintain competitiveness in a global market and making them feel capable of dealing with temporary and future developments—new psychological contracts created by individuals will likely increase their wellbeing (Lent et al., 2011; Ahmed and Nawaz, 2015; Akgunduz et al., 2018). In addition, individuals can process the same things and tasks more efficiently and in less time with relevant experience, updated skills, and knowledge (Lent et al., 2011; Chang and Edwards, 2015) - as well as a well-developed social network—so as to improve EE. The abundance of time saved will be used for life needs and personal future planning, thereby enhancing SWB (Thompson et al., 2016). Similarly, employees with higher employability can face the job challenges of the future with a broader perspective, particularly when they face the challenge of the COVID-19 pandemic. In addition to mastering the content of an organizational task, they also have a more precise direction for planning and preparing to achieve the task (Ineson et al., 2013; Chang and Edwards, 2015), reducing their insecurity and enhancing SWB. Based on the above phenomena, another hypothesis of this study is as follows:

*H2: EE has a positive and significant impact on employees’ SWB.*

### Self-Efficacy

Social Cognitive Career Theory scholars argue that individuals’ behavioral outcomes will be influenced by both environmental and cognitive factors in a given situation, especially beliefs that lead to success and behavior changes (Brown et al., 2011; Chin and Rasdi, 2014; Chang and Edwards, 2015; Liguori et al., 2019). They call these beliefs “self-efficacy,” an important cognitive variable in personal factors during the process of interpreting individual formative behaviors (Caesens and Stinglhamber, 2014), and interaction with the environment (Lent et al., 2011; Duffy et al., 2014; Chang and Edwards, 2015; Jemini-Gashi et al., 2019). It can also be seen as the basis for human behavioral motivation (Cordova et al., 2014), mental health, and personal achievement (Lent et al., 2011; Liguori et al., 2019). Self-efficacy is widely used in the field of human resources to explore the psychological cognitive factors of employees of different situations and their positive impact on task achievement and employee career development (Brown et al., 2011; Caesens and Stinglhamber, 2014; Duffy et al., 2014; Jemini-Gashi et al., 2019).

According to the above discussion, employees who have confidence in their abilities will have more efficient behaviors and better interpersonal relationships than those who do not (Brown et al., 2011; Chin and Rasdi, 2014; Chang and Edwards, 2015). According to Chin and Rasdi (2014), highly self-motivated employees look for resources and opportunities to accomplish tasks that exist in social networks (Lent et al., 2011; Thompson et al., 2016). Only by establishing and maintaining network relationships can they achieve their goals. Knowledge and resources are needed (Lent et al., 2011; Jemini-Gashi et al., 2019). Furthermore, teamwork can also be seen as a strong network relationship, and the process of employees solving problems and achieving tasks through teamwork will positively affect their EE (Duffy et al., 2014; Chang and Edwards, 2015). According to the above, this study proposes the following hypothesis:

*H3: Self-efficacy has a positive and significant impact on EE.*

Some scholars have focused their investigations on mental health concerns, POS (Chin and Rasdi, 2014), and life styles in employees (Lent et al., 2011). However, few studies thus far have tapped into this population’s general self-efficacy and SWB. Jemini-Gashi et al. (2019) indicated in their research findings that individuals reported a lower level of support, limited sources of support, and low perceived support (Brown et al., 2011). In other words, according to Caesens and Stinglhamber (2014), employees with a high level of self-efficacy are likely to obtain a variety of benefits at work that ultimately lead to a higher level of job satisfaction. It implies that employees’ general self-efficacy and SWB decrease because they are unable to receive timely and necessary psychological support when confronting work stress (Thompson et al., 2016). In addition, it might contribute to unique stressors. With the outbreak of COVID-19, people often suffer from a series of irrational emotions, such as anxiety, and are susceptibility to stress (Clauw et al., 2003). When employees have a high sense of self-efficacy, their mental resilience and recovery capability are stronger, and they have a stronger risk tolerance. Conversely, employees with higher self-efficacy have higher SWB. In summary, the study infers the following:

*H4: Self-efficacy has a positive and significant impact on employees’ SWB.*

### Developing Subjective Wellbeing in Human Resources

Two causal mechanisms contribute to SWB development in human resources: prior knowledge (PK) and perceived organizational support (POS). In the case of wellbeing building support, organizations or supervisors can devise the organizational context, such as individual and organizational factors (Chin and Rasdi, 2014; Chang and Edwards, 2015; Thompson et al., 2016; Liguori et al., 2019) to enhance the efficiency and responsiveness of knowledge gain. Scholars claim that organizations or supervisors utilize, integrate, and reconfigure individual and organizational factors to building an optimal organizational context for constructing employees’ SWB (Lent et al., 2011; Chin and Rasdi, 2014; Ahmed and Nawaz, 2015; Kurtessis et al., 2017; Akgunduz et al., 2018). Organizations or supervisors can implement a series of support activities to pinpoint individual and organizational factors (Thompson et al., 2016; Liguori et al., 2019), where PK focuses on sensing internal existed knowledge and skills (Ineson et al., 2013; Williams and Lombrozo, 2013; Cordova et al., 2014; Li et al., 2015; Hajizadeh and Zali, 2016) and POS on providing tangible and intangible resources for facilitating employees’ ability to achieve their tasks or goals (Caesens and Stinglhamber, 2014; Ahmed and Nawaz, 2015; Lamm et al., 2015; Liguori et al., 2019). This study considers a better way to build SWB in facilitating the adaptation of support activities for PK and POS.

#### Wellbeing Building Support Mechanism: Prior Knowledge (PK)

The explanation of people in the current situation and information depends on self-perception. By self-perception, people can identify things and the environment they are in Chang and Edwards (2015). In other words, self-perception helps learners in learning, but the learners may not know it (Ineson et al., 2013; Thompson et al., 2016). The function of the prior capability is to help the learner understand external knowledge and information and then combine the acquired knowledge intention with the prior capability of the learner (Williams and Lombrozo, 2013; Li et al., 2015), thus generating a more enriched basis of prior capabilities (Ineson et al., 2013; Cordova et al., 2014). Therefore, prior capability is not unchanging, but can increase as time goes by, showing a characteristic of path dependency (Williams and Lombrozo, 2013; Li et al., 2015), and the PK can be strengthened based on the learning attitude and motivation of the learner (Ineson et al., 2013; Cordova et al., 2014; Hajizadeh and Zali, 2016; Liguori et al., 2019).

In studies of PK, scholars have discussed the effect of PK based on different theories (Cordova et al., 2014; Hajizadeh and Zali, 2016). Although some empirical studies indicated that PK has no effect on employee performance, a few scholars still believe that PK has a significant correlation with learning (Cordova et al., 2014). By reference to the cognitive load theory, Amadieu et al. (2009) have studied the effect of staff PK in learning internal electronic documents in the organization (Williams and Lombrozo, 2013). They reached a conclusion that the staff with a high degree of PK were more competent in processing information and organizing their study route using their own mental model (Williams and Lombrozo, 2013; Hajizadeh and Zali, 2016; Liguori et al., 2019). Besides, staff with a high degree of PK were very unlikely to suffer from work confusion compared to those with a low degree of PK (Ineson et al., 2013; Liguori et al., 2019). The possible reason is that the explicit and written knowledge has a limited effect, even if the staff have a high degree of PK in this regard (Williams and Lombrozo, 2013; Hajizadeh and Zali, 2016); but the implicit and complex knowledge will drive employees with a high degree of PK of this kind to look carefully and deeply into knowledge intention (Cordova et al., 2014), which is conducive to transforming this exploring process into their own EE. To sum up, this study proposes another hypothesis as follows:

*H5: PK has a positive and significant impact on EE.*

Employees with more PK facilitate themselves to assess more external knowledge to solve work problems and challenges, thus achieving individual goals and enhancing the personal sense of achievement (Williams and Lombrozo, 2013; Cordova et al., 2014). In other words, in the task implementation process, employees enhancing their own competence through learning, sensing, and integrating various knowledge, have more PK (Ineson et al., 2013; Hajizadeh and Zali, 2016). This is conducive to improving personal feelings of SWB. With more PK, employees will identify valuable and useful information and knowledge to deal with business enquiries from an external environment, thus affecting job satisfaction and efficiency. Some previous studies indicated that it could be reasonable to expect immediate significant self-efficacy change (Ineson et al., 2013; Liguori et al., 2019), coupled with a significant improvement of PK over time for employees (Cordova et al., 2014). Likewise, employees can mitigate the influence caused by bad environmental events using their own accumulated knowledge or resources in the face of negative environmental events or when in need of assistance (Hajizadeh and Zali, 2016). When employees feel greatly stressed, and importance resources are lost, employees’ estimation of the stress scenario will be affected if they have enough PK, thus reducing adaptive strategies for negative emotions and improper use (Ineson et al., 2013). Therefore, this study proposes H6:

*H6: PK has a positive and significant impact on employees’ self-efficacy.*

#### Wellbeing Building Support Mechanism: Perceived Organizational Support (POS)

Perceived organizational support (POS), occasionally used interchangeably with POS (Ahmed and Nawaz, 2015; Akgunduz et al., 2018), is how employees perceive whether an organization cares about their wellbeing and contributions (Gillet et al., 2012; Caesens and Stinglhamber, 2014; Demir, 2015) or whether the organization helps them achieve professional and personal goals (Uppal and Mishra, 2014; Kurtessis et al., 2017; Liguori et al., 2019). When employees perceive good organizational support, they also feel safer in their jobs and are engaged in their work (Kose, 2016; Kurtessis et al., 2017). POS has been strongly correlated with many positive workplace characteristics and behaviors, such as a positive organizational climate (Ahmed and Nawaz, 2015; Kose, 2016; Jemini-Gashi et al., 2019) and positive organizational citizenship behavior (Caesens and Stinglhamber, 2014; Demir, 2015; Lamm et al., 2015; Akgunduz et al., 2018). Many of these associations appear to be related to other variables within this study (Meyers et al., 2019). For instance, Kose (2016) discusses organizational citizenship behavior as a willingness of employees to help others beyond the scope of their assigned duties, which appears to be similar to a social dimension in self-efficacy and EE.

Previous studies have indicated a significant relationship between POS and self-efficacy (Caesens and Stinglhamber, 2014; Kose, 2016). When workers feel as though the organization is concerned about their wellbeing, they, in turn, offer their dedication as a social exchange. POS also boosts employees’ sense of belonging (Demir, 2015; Lamm et al., 2015; Akgunduz et al., 2018). About the relationship between POS and self-efficacy, Kose (2016) identified that employees who perceive organizational support often feel secure in their positions and believe that their organizations are concerned about their professional development (Lent et al., 2011; Uppal and Mishra, 2014; Schultz et al., 2015; Kurtessis et al., 2017). It stands to reason that workers who believe that their organizations care about their personal and professional life would be willing to seek out more resources for task completion or to gain more responsibilities (Akgunduz et al., 2018), which are dimensions of self-efficacy and EE (Lent et al., 2011; Caesens and Stinglhamber, 2014). POS has a positive association with organizational citizenship behavior (Demir, 2015; Meyers et al., 2019), which predicts more helping behaviors within an organization.

Perceived organizational support is the most direct and effective support source for employees (Akgunduz et al., 2018). Being organized would assist employees in job demands and solve confusion and anxiety arising from the application of technological tools at work (Lent et al., 2011; Lamm et al., 2015). Besides, the support for effective working through organization will improve the status of working engagement and perfect employees’ successful task achieving ability (Kurtessis et al., 2017; Jemini-Gashi et al., 2019; Liguori et al., 2019). According to Akgunduz et al. (2018), sufficiently competent and motivated employees can achieve their organizations’ goals and perform as required without managerial supervision (Meyers et al., 2019). POS is also related to theories of social exchange (Ahmed and Nawaz, 2015; Kurtessis et al., 2017). Combined with psychological features of employees, conducive working environments can be created to enable employees to be more confident in completing job tasks (Caesens and Stinglhamber, 2014; Liguori et al., 2019). Employees will be more driven and motivated to engage in job objects and understand values and insights brought on by achieving tasks and solving problems (Lent et al., 2011; Ahmed and Nawaz, 2015), thus improving employee self-efficacy, if they feel the positive psychological environment established by POS from supervisors and peers. Therefore, this study proposes H7:

*H7: POS has a positive and significant impact on employees’ self-efficacy.*

Moreover, POS, with its relationship with EE, is helpful in improving employees’ interest in work and the application of their professional skills (Caesens and Stinglhamber, 2014; Ahmed and Nawaz, 2015), and in further enhancing employees’ capability (Lent et al., 2011; Liguori et al., 2019). When facing practical problems, such as critical analysis, problem solving, and reflection, employees can demonstrate better working attitudes and critical thinking ability (Schultz et al., 2015; Jemini-Gashi et al., 2019). Akgunduz et al. (2018) claimed that the support employees receive from supervisors or organizations increases employee creativity, thus improving their employment skills (Gillet et al., 2012; Caesens and Stinglhamber, 2014). Mulholland and O’Connor (2016) have confirmed that employees who have accepted the POS pattern will change their working skills, attitudes, and behaviors so as to enhance their critical thinking, autonomy, and employment-related competencies. Therefore, this study proposes H8:

*H8: POS has a positive and significant impact on EE.*

**FIGURE 1:**
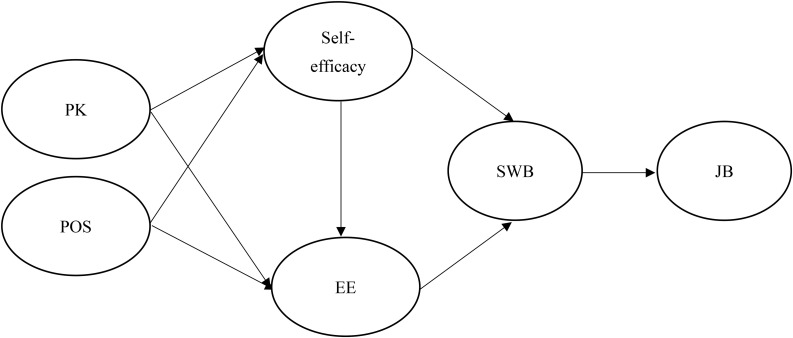
Research framework.

Based on the above hypotheses, this study proposes the following research framework Figure 1.

## Methodology

### Sampling

The research sample in this study comprised employees. Purposive sampling was adopted. To understand whether the subject attributes would influence the research results, non-response bias tests were verified. The results indicated that subject did not significantly impact the research variables, so did not need to be included as an independent variable in subsequent analyses. To discuss the impact of negative factors brought by COVID-19 on employees, regions with different levels of isolation have been selected for comparison to verify the correlation among various factors in the model of SCCT proposed in this study. Taiwan and mainland China have a great degree of similarity in cultural and work activities, but employees in the two regions have a different awareness of virus threat and epidemic prevention due to differences in isolation level, which further affects their social-cognition factors, thus Taiwanese and mainland China companies are adopted as the sample. A structural model has been analyzed in this study, and structural equation modeling (SEM) has been adopted to verify our research framework. Scholars have argued that the sample size needed for SEM analysis should be at least 200 (MacCallum et al., 1996), while Hair et al. (2009) suggested that the sample size should be more than 500 when there are many latent variables in the model. This study proposed a framework to explore the correlations and development mode of PK, POS, self-efficacy, SE, SWB, and JB. This study selected more than 10 Taiwanese and mainland China companies, and then sent 2,000 questionnaires to each of them. After sampling, a total of 623 Taiwanese questionnaires and 513 mainland China questionnaires were returned, for an effective response rate of 62.3% and 51.3%.

### Measures

All constructs were measured by multiple-item scales based on previous studies. The construct of PK adopted the scale proposed by Silva et al. (2013), including 10 items. The construct of POS was divided into supervisor and colleague support (4 items) and organizational support (8 items). This study adopted the scales proposed by De Vos et al. (2011). Similar to the EE scale reported by Pan and Lee (2011), 18 items were used to capture general ability for work (GAW) (8 items), professional ability for work (PAW) (4 items), attitude at work (AW) (3 items), and career planning and confidence (CPC) (3 items). For self-efficacy, the scale was revised and integrated with six items with higher reliability and validity by Rigotti et al. (2008) based on the self-efficacy scale developed by Schyns and von Collani (2002). Subjective wellbeing was measured using Keyes’s (2005) subjective wellbeing instrument (adolescent version), which comprehensively assesses wellbeing in terms of emotional (3 items), psychological (4 items), and social (4 items) dimensions. For JB, five items were selected on the basis of Janssen (2001) scale. All items were measured with a five-point Likert scale (1 = totally disagree; 5 = totally agree).

### Data Analysis Strategy

Research tools are distinguished in this study to achieve rigorous analysis results for the research framework and to correspond with the contents to be analyzed in issues of this study. Measurement model and structural model are used in this study. In the measurement model, AMOS 23.0 is applied for the confirmatory factor analysis (CFA) to verify the convergent validity and discriminant validity of the scale. In addition, scholars hold that PLS-SEM is more competent than CB-SEM in estimating much more complex models with smaller sample sizes (Shiau and Chau, 2016; Hair et al., 2019; Khan et al., 2019; Shiau et al., 2019). Compared with CB-SEM, PLS-SEM is more suitable for this study in the following cases: the research objective is exploratory research for theory development; the analysis is conducted for a prediction purpose; the structural model is complex; the structural model includes one or more formative constructs; distribution has a lack of normality; and research requires latent variable scores for consequent analyses (Gefen et al., 2011; Shiau and Chau, 2016; Hair et al., 2019; Khan et al., 2019; Shiau et al., 2019). Thus, in the structural model, this study adopts Smart-PLS for PLS-SEM to verify the hypotheses and comparative analysis of this study.

## Results

### Evaluation of the Measurement Model

All scales used in this study were found to be reliable, with Cronbach’s α ranging from 0.83 to 0.96. Table 1 shows the reliability of each scale, and the factor loadings for each item therein. In order to gauge validity, this study employed confirmatory factor analysis (CFA) using AMOS 23.0 to verify the construct validity (both convergent and discriminant) of the scales. Two samples from different regions collected based on the isolation level of the pandemic have been respectively tested by CFA. In the following Table 1, the correlation coefficient in the upper triangle is the mainland China sample, while the correlation coefficient in the lower triangle is the Taiwanese sample. According to Hair et al.’s (2009) recommended validity criteria, CFA results show standardized factor loading of higher than 0.5; average variance extracted (AVE) ranges between 0.539 and 0.729; and composite reliability (CR) ranges between 0.800 and 0.918. All three criteria for convergent validity were met, and correlation coefficients were all less than the square root of the AVE within one dimension, suggesting that each dimension in this study had good discriminant validity.

**TABLE 1:** Measurement properties.

		1	2	3	4	5	6	7	8	9	10	11	12
(1) PK		0.79/0.82	0.284	0.290	0.084	0.089	0.630	0.711	0.838	0.162	0.185	0.223	0.240
(2) Organization		0.479	*0.81/0.72*	0.713	0.458	0.126	0.188	0.288	0.291	0.585	0.654	0.708	0.718
(3) Supervisor		0.520	0.894	*0.86/0.78*	0.445	0.113	0.179	0.284	0.297	0.654	0.713	0.736	0.750
(4) Self-efficacy		0.533	0.600	0.665	*0.82/0.71*	0.103	0.102	0.134	0.116	0.323	0.342	0.368	0.334
(5) GAW		0.552	0.420	0.460	0.504	*0.78/0.76*	0.123	0.135	0.103	0.048	0.108	0.109	0.059
(6) PAW		0.470	0.373	0.424	0.501	0.814	*0.85/0.83*	0.806	0.598	0.144	0.115	0.154	0.176
(7) AW		0.527	0.436	0.508	0.558	0.757	0.746	*0.85/0.82*	0.684	0.220	0.181	0.239	0.226
(8) CPC		0.543	0.463	0.498	0.562	0.658	0.644	0.743	*0.89/0.89*	0.194	0.184	0.221	0.250
(9) Emotional		0.433	0.535	0.604	0.578	0.414	0.397	0.491	0.449	*0.92/0.85*	0.762	0.639	0.621
(10) Psychological		0.512	0.588	0.657	0.689	0.515	0.496	0.532	0.522	0.792	*0.86/0.81*	0.828	0.654
(11) Social		0.448	0.582	0.647	0.633	0.486	0.447	0.521	0.507	0.698	0.737	*0.88/0.85*	0.697
(12) JB		0.501	0.572	0.628	0.691	0.511	0.465	0.516	0.493	0.554	0.605	0.540	*0.77/0.79*
Mean	Taiwan	3.413	3.515	3.533	3.753	3.533	3.638	3.604	3.557	3.632	3.710	3.523	3.581
	China	3.975	4.292	4.304	3.922	3.688	3.834	3.878	3.964	4.369	4.450	4.555	4.400
SD	Taiwan	0.658	0.708	0.671	0.624	0.640	0.701	0.703	0.722	0.719	0.688	0.780	0.566
	China	0.666	0.544	0.472	0.408	0.621	0.714	0.686	0.707	0.534	0.517	0.542	0.538
A	Taiwan	0.934	0.926	0.883	0.903	0.905	0.877	0.801	0.863	0.905	0.887	0.901	0.750
	China	0.946	0.865	0.778	0.773	0.741	0.846	0.749	0.876	0.800	0.827	0.867	0.844
AVE	Taiwan	0.628	0.660	0.740	0.674	0.604	0.731	0.718	0.785	0.841	0.747	0.772	0.590
	China	0.679	0.521	0.604	0.500	0.581	0.685	0.668	0.801	0.717	0.661	0.715	0.617
CR	Taiwan	0.944	0.939	0.919	0.925	0.924	0.916	0.884	0.916	0.941	0.922	0.931	0.856
	China	0.955	0.895	0.858	0.837	0.719	0.897	0.857	0.924	0.883	0.886	0.909	0.889

*Square root of AVE for each latent construct is given in diagonals.*

### Inner Model Analysis

Prior to hypotheses testing, the values of the variance inflation factor (VIF) were determined. The VIF values were less than 5, ranging from 1 to 1.857. Thus, there were no co-linearity issues among the predictor latent variables (Hair et al., 2017).

**FIGURE 2:**
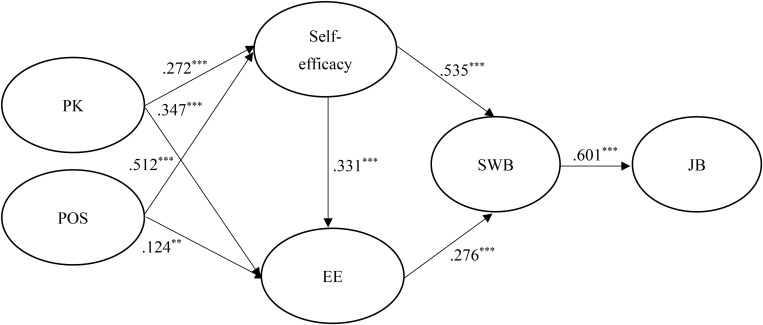
Structural model on Taiwanese employees. ***p < 0.001.

**FIGURE 3:**
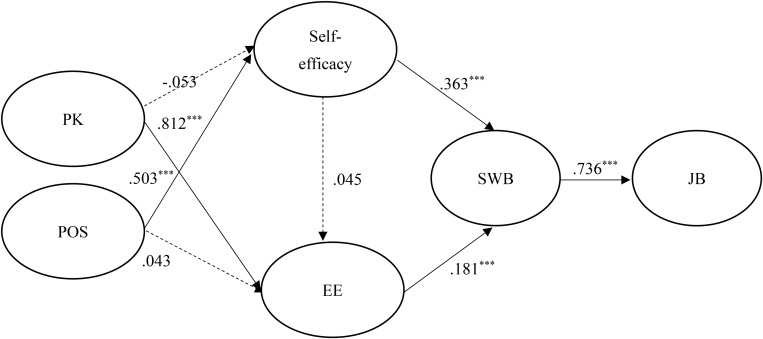
Structural model of mainland China employees.

Figures 2, 3 show the results of the hypothesized relationships and standardized coefficients in Taiwanese and mainland China samples. The results showed that SWB was positively and significantly related to JB (β_Taiwan_ = 0.601, *p* <0.001; β_China_ = 0.736, *p* <0.001), supporting H1. Self-efficacy (β_Taiwan_ = 0.535, *p* <0.001; β_China_ = 0.363, *p* <0.001) and SE (β_Taiwan_ = 0.276, *p* <0.001; β_China_ = 0.181, *p* <0.001) were also positively and significantly related to SWB, supporting H2 and H4. In addition, self-efficacy (β_Taiwan_ = 0.331, *p* <0.001; β_China_ = 0.045, *p* > 0.1) was positively and significantly related to SE in the Taiwanese sample rather than the mainland China sample, partially supporting H3. Similarly, the paths of PK → self-efficacy (β_Taiwan_ = 0.272, *p* <0.001; β_China_ = -0.053, *p* > 0.1) and POS → EE (β_Taiwan_ = 0.124, *p* <0.01; β_China_ = 0.043, *p* > 0.1), showed that the relationships were positive and significant in the Taiwanese sample rather than the mainland China sample, therefore partially supporting H6 and H8. Finally, the paths of PK → SE (β_Taiwan_ = 0.347, *p* <0.001; β_China_ = 0.812, *p* <0.001) and POS → self-efficacy (β_Taiwan_ = 0.512, *p* <0.001; β_China_ = 0.503, *p* <0.001) showed that the relationships were positive and significant in both samples, supporting H5 and H7. The Stone-Geisser Q2 values obtained through the blindfolding procedures for self-efficacy (Q^2^ = 0.193), EE (Q^2^ = 0.340), SWB (Q^2^ = 0.344) and JB (Q^2^ = 0.342) were larger than zero, supporting the predictive relevance of the model (Hair et al., 2017). Finally, the standardized root mean square residual value for the structural model was < 0.08 (0.062 for our model), which indicated good model fit (Hair et al., 2017).

### Multiple Group Analysis (MGA): Taiwan and Mainland China

It was confirmed that the measurement pattern was stable. However, in order to avoid overgeneralizing the data-driven patterns and theories, the study followed the suggestion of Hair et al. (2010) and divided the sample data into two groups based on regions (623 Taiwanese and 513 mainland China employees, respectively). The partial measurement invariance was established, which was the basic requirement to compare as well as interpret the PLS-SEM’s findings for examining the specific MGA group’s differences (Henseler et al., 2016). Table 2 indicates the structural models’ results and MGA by using non-parametric methods including Henseler’s MGA as recommended by Henseler et al. (2009). To highlight the impact brought by isolation levels during the COVID-19 pandemic, the path coefficients between the variables of the SCCT model in different regions were compared. Despite several differences in terms of significant path estimates between the groups, as indicated in Table 2, the multi-group permutation tests (final column on the right) showed that there were seven significant differences between the two groups on all the paths. Specifically, in the structural model of Taiwanese employees, all paths had significantly positive effects. However, compared to the structural model of mainland China employees, PK and POS appeared to have no significant effects on self-efficacy and EE. This suggests that the Taiwanese employees achieved greater SWB development from having well-established PK and POS. It was found from the comparative analysis of path coefficients that the isolation level during the COVID-19 pandemic could lead to differences in employees’ cognition of career development in different regions.

**TABLE 2:** Multi-group analysis result.

Path	Path coefficients (confidence interval)		—β_Taiwan_-β_China_—	*p*-value
			
	β_Taiwan_ (2.5%-97.5%)	β_China_ (2.5%-97.5%)		Henseler’s MGA
H1: SWB → JB	0.601 (0.533 -0.655)	0.736 (0.688 -0.774)	0.135	0.000
H2: Self-efficacy → SWB	0.535 (0.455 -0.608)	0.363 (0.276 -0.437)	0.172	0.049
H3: Self-efficacy → EE	0.331 (0.246 -0.406)	0.045 (-0.009 - 0.107)	0.287	0.000
H4: EE → SWB	0.276 (0.190 -0.360)	0.181 (0.109 -0.253)	0.095	0.000
H5: PK → EE	0.347 (0.244 -0.437)	0.812 (0.772 -0.845)	0.465	0.000
H6: PK → self-efficacy	0.272 (0.200 -0.349)	-0.053 (-0.145 -0.025)	0.325	0.000
H7: POS → EE	0.124 (0.041 -0.207)	0.043 (-0.019 -0.106)	0.082	0.061
H8: POS → self-efficacy	0.512 (0.424 -0.584)	0.503 (0.423 -0.581)	0.009	0.431

## Discussion and Conclusion

Based on the isolation level during the COVID-19 pandemic, this study analyzed the psychological cognitive status of employees in different regions in the work environment when they faced the pandemic, put forward variable sources to be verified with SCCT, and established a complete conceptual framework. This study took Taiwanese and mainland China employees as research samples to test the PK, POS, self-efficacy, SE, SWB, and JB correlation using SCCT. This study will fill the theoretical gap in the application of Western theories under the Eastern context (Lent et al., 1994; Brown et al., 2011; Chang and Edwards, 2015), and increase the generalization of the theory. Based on our research findings, this study aimed to provide the following contributions. First, there are few studies to verify employees’ SWB based on a huge environmental challenge (Thompson et al., 2016). This study investigated employees’ competence enhancement process and SWB in the situation of the COVID-19 global pandemic and attempted to offer practical implications for company administrations. Second, most previous studies on SCCT explored the importance of environmental factors (Brown et al., 2011; Hansen et al., 2012; Duffy et al., 2013; Chang and Edwards, 2015; Liguori et al., 2019) but only a few studies provided essential contributions with global environmental factors. In this study, the global pandemic of COVID-19 was adopted as a recessive moderator to verify the theoretical development of SCCT in the face of major global environmental issues, and further fill the theoretical gap and enrich the theoretical foundation of SCCT. Third, in addition to verifying the research framework built through SCCT in an Asian context, this study also included different perspectives of working environments (online vs. field working). Our findings will provide more insights and suggestions in terms of human resource theories.

The results indicated that the PK and POS of Taiwanese employees were positively related to their self-efficacy and EE, whereas there were no significant effects on paths of PK → self-efficacy and POS → EE on mainland China employees. These results correspond with those of Hansen et al. (2012), Lent et al. (2016), and Meyers et al. (2019); on the basis of SCCT, they believe that the environmental differences influence employees’ working status and attitudes (Rehg et al., 2012), causing competence and skills-gaining to differ. Our findings were largely consistent with those of these prior studies, supporting the SCCT model’s availability across a range of regions (Hansen et al., 2012). Besides, there may be insignificant correlations between paths of PK → self-efficacy and POS → EE on mainland China employees because under strict isolation policy, mainland China employees found it hard to acquire sufficient psychological support from their organization (Schultz et al., 2015). Supervisors or colleagues led by economic activity stagnation foster suitable EE and confidence to achieve tasks. Relatively speaking, Taiwanese employees who faced a low level of isolation during the pandemic situation had access to more sources of support with the guidance of isolation policy, which was more conducive to the accumulation of psychological capital when they faced the threat of the pandemic. Moreover, the results showed positive correlations among paths of PK → EE and POS → self-efficacy for both Taiwanese and mainland China employees. It is also worth noting that the individual and organizational support mechanism implied that employees with more PK and POS from their organization or supervisors were likely to be more involved in the working environment and actively participate in task activities, thus obtaining the ability and confidence to achieve tasks, such as the development of systematic/integrative thinking and problem-solving skills. This finding is consistent with the findings of a number of previous studies (Schultz et al., 2015) supporting the relationship between support mechanism and self-efficacy. Although researchers have begun to examine the link among POS, work conditions and work motivation according to motivation theory (e.g., Schultz et al., 2015), few previous studies to the best of our knowledge have investigated the influence of individual or organizational factors on psychological and competence needs in the context of a global pandemic. The present research is thus the first to demonstrate that the more employees perceive high levels of SWB building mechanism (Gillet et al., 2012), the more they will satisfy their self-efficacy and EE.

Besides, the findings show that self-efficacy and SE are strong contributors to SWB for both Taiwanese and mainland China employees. Furthermore, self-efficacy plays a key mediating role in the research model of SCCT. In strict isolation, employees tend to have work powerlessness, job insecurity, stress, and other factors when facing the pandemic, which causes employees of mainland China to fail to enhance their EE through self-efficacy. These findings are quite consistent with those of Lent et al. (2016) and Meyers et al. (2019), who verified the wellbeing model cross-sectionally in different samples of employees (Hansen et al., 2012). Moreover, different from the study of Meyers et al. (2019), this study compares samples of different regions in the same model, such as Germany, Indonesia, the Netherlands, Romania, and South Africa, reports good overall model-data fit in both samples (Taiwan and mainland China), and verifies direct and indirect effects of self-efficacy generated in the wellbeing model of SCCT on SWB. However, differing from the studies of Lent et al. (2016) and Meyers et al. (2019), this study also considers psychological effects of global environmental events, and enriches the theoretical model and SCCT of wellbeing based on the region analysis. This study has further verified that the expectation and desire for wellbeing from employees in different regions under the pressure of isolation in a pandemic can effectively provide them with relief from the stress and uncertainty of various negative factors arising from the pandemic. Moreover, the results indicated that SWB was found to be positively and significantly related to JB for both Taiwanese and mainland China employees. This finding implies that a positive psychological attitude significantly facilitates employees to improve their JB in different working environments, specifically when enduring a tough situation. The positive influence of SWB on JB is in line with the findings of previous studies, which may improve the explanatory utility and cultural relevance of SCCT models for individuals who reside in different countries and cultures.

The study also made a theoretical contribution by examining the extent to which employees’ regions (Taiwan and mainland China) influences the relationships among POS, PK, self-efficacy, EE, SWB, and JB. This is consistent with recent work by Sheu and Bordon (2017) showing that contextual supports have received more attention in international SCCT research. The geographic distribution of international SCCT research showed that more empirical attention is still needed in Asian and European countries. Sheu and Bordon (2017) also suggested that cross-regions differences should be included and discussed in future research. Since there are policies for different levels of isolation led by the COVID-19 pandemic, such environmental conditions affect the differences in the psychological cognition of employees in different regions and indirectly lead to the differences in the structural model of SCCT. Examining the structural model across two groups, it was predicted that the structural relationships among the constructs would be stronger for multinational enterprises managers with Taiwanese and mainland China employees. However, the PLS-SEM multi-group analysis showed the working environment as a moderator variable, indicating that the presence of an offline office did strengthen the relationships between PK, POS, self-efficacy, SE, SWB, and JB. In other words, the operation of factors in the SCCT model for the Taiwanese employees in a lower level of isolation shows a more significant leverage effect than that of the mainland China employees in isolation of a higher level; similarly, the results imply the significance of social interaction in SCCT when an unpredictable pandemic occurs.

### Practical Implications

In summary, according to our findings, this study suggests some important practical implications for improving the quality of human resources. Firstly, in this study, POS and PK were perceived as equally important and predictive of employees’ own perceived levels of self-efficacy, EE, thus affecting SWB. Individual and organizational building mechanisms of mentality will contribute to employees obtaining more resource and psychological support, which are essential conditions for improving SWB. Thus, at the present stage when countries and regions all over the world combat COVID-19, in face of similar events, organizations should encourage supervisors to actively form close ties with employees, build communication platforms using technological media and information technology tools, and provide task or psychological support in real time.

Second, external environment factors, especially the global epidemic COVID-19, may affect employees’ working status. Thus, managers must be examined for a sense of risk management. On this basis, this study suggests companies or organizations to take preventive risk management measures to tackle threats and challenges brought on by adaptive risks in the face of similar events. Although this event caused all employees to take up online working, not all employees were equipped with the required technological media or information technology tools. In consequence, managers should count up the number of employees who have information technology tools first and measure whether tasks or work are able to be done online; and the tasks or work that are not suitable for online working should be adjusted in terms of schedule.

Third, in light of the structural patterns of the two regions, SWB deriving from self-efficacy of Taiwanese employees is superior to that of employees in mainland China. It can be seen that working online or not both have an effect on employees. Employees in regions that are blocked for a longer time tend to feel more helpless, incapable, and anxious. Even if employees have confidence in completing tasks, they are affected by negative energy caused by blockage. This study suggests that managers may offer other kinds of support, such as opportunities, resources, and autonomy to help employees to overcome the environmental threats and challenges and engage in improving their wellbeing.

### Research Limitations

The research results contribute to the literature on region-specific employees, SCCT, and employee wellbeing; nevertheless, some limitations still exist and represent further research directions. First, SCCT has obtained considerable status in the psychological field, but only a few studies have considered the relationship between building mechanism and wellbeing of employees. Although the building mechanism was constructed with reference to SCCT in this study, and important organizational theories can be derived from the research results, other motivation theories, such as organizational learning theory, self-efficacy theory, and hierarchy needs theory, still apply to explain how to trigger SWB in region-specific employees. Thus, it is suggested that future research should utilize different theoretical models in order to identify relevant psychological dimensions influencing employees’ wellbeing. Second, this study required employees to self-report details on their psychological building mechanism as the indicator, mainly because the actual data are confidential and not easily obtained. However, errors may exist in the employees’ self-statement of their psychological status. The link between building mechanism and wellbeing may be better understood if employees’ actual psychological status is assessed, with due consideration for research ethics. Besides, this study suggests that future researchers include interview content and employees’ observations of working status in their studies to support the research results and make a comprehensive judgement. Third, due to restrictions of time and space, 1136 valid questionnaires in total were sampled in this study. The research objects were divided into Taiwanese and mainland China employees. Future research could explore and compare other groups, in addition to expanding the quantity of samples and improving the research representativeness, so as to provide additional insights relevant to organizational behavior management.

## Data Availability Statement

The raw data supporting the conclusions of this article will be made available by the authors, without undue reservation.

## Ethics Statement

The studies involving human participants were reviewed and approved by Ethics Committee in University of Taipei. The patients/participants provided their written informed consent to participate in this study. Written informed consent was not obtained from the individual(s) for the publication of any potentially identifiable images or data included in this article.

## Author Contributions

This study is a joint work of the three authors. MY-PP and T-CL contributed to the ideas of research, collection of data, and empirical analysis. MY-PP, LW, and H-KH contributed to the data analysis, design of research methods, and tables. MY-PP, LW, and H-KH participated in developing a research design, writing, and interpreting the analysis. All authors contributed to the literature review and conclusions.

## Conflict of Interest

The authors declare that the research was conducted in the absence of any commercial or financial relationships that could be construed as a potential conflict of interest.
