# Corrigendum: Factors Influencing Employees' Subjective Wellbeing and Job Performance During the COVID-19 Global Pandemic: The Perspective of Social Cognitive Career Theory

**DOI:** 10.3389/fpsyg.2021.679600

**Published:** 2021-08-09

**Authors:** Tzai-Chiao Lee, Michael Yao-Ping Peng, Lin Wang, Hao-Kai Hung

**Affiliations:** ^1^Department of Accounting and Auditing, Guangxi University of Finance and Economics, Nanning, China; ^2^School of Economics & Management, Foshan University, Foshan, China; ^3^Department of Business Administration, Yango University, Fuzhou, China

**Keywords:** prior knowledge, perceived organizational support, self-efficacy, employee employability, subjective well-being, job performance

In the original article, there was an error in the author list as published. After a unanimous decision, the authors consider that Din Jong had a low contribution to this article and does not qualify for authorship of this article. Therefore, Din Jong was removed from the author list.

## Author Contributions

This study is a joint work of the three authors. MY-PP and T-CL contributed to the ideas of research, collection of data, and empirical analysis. MY-PP and LW contributed to the data analysis, design of research methods, and tables. MY-PP, LW, and H-KH participated in developing a research design, writing, and interpreting the analysis. All four authors contributed to the literature review and conclusions.

The authors apologize for this error and state that this does not change the scientific conclusions of the article in any way. The original article has been updated.

## Publisher's Note

All claims expressed in this article are solely those of the authors and do not necessarily represent those of their affiliated organizations, or those of the publisher, the editors and the reviewers. Any product that may be evaluated in this article, or claim that may be made by its manufacturer, is not guaranteed or endorsed by the publisher.

